# Caloric restriction effects on liver mTOR signaling are time-of-day dependent

**DOI:** 10.18632/aging.101498

**Published:** 2018-07-16

**Authors:** Richa Tulsian, Nikkhil Velingkaar, Roman Kondratov

**Affiliations:** 1 Department of Biological, Geological, and Environmental Sciences and Center for Gene Regulation in Health and Diseases, Cleveland State University, Cleveland, OH 44115, USA

**Keywords:** metabolism, mTOR signaling pathway, aging, glucose, biological clocks, circadian clocks, insulin sensitivity, protein translation

## Abstract

The regulation of mechanistic target of rapamycin (mTOR) signaling contributes to the metabolic effects of a calorie restriction (CR) diet. We assayed the effect of CR on the activity of mTOR complex 1 (mTORC1) and mTOR complex 2 (mTORC2) in the liver of mice at six different times across the day. CR effects on mTORC1 and mTORC2 activities were time-of-day dependent. CR induced mTORC1 activity at one time, reduced at two times and has no effect during other times. CR induced mTORC2 activity at one time of the day and has no effects at other times. Circadian clocks are implemented in the regulation of mTOR signaling in mammals and mechanisms of CR. We assayed the effect of CR on mTOR signaling in the liver of mice deficient for circadian transcriptional regulators BMAL1 and CRYs. The CR induced suppression of mTORC1 activity was observed in both clock mutants, while up regulation of mTORC2 was observed in the liver of CRY deficient but not in the liver of BMAL1 deficient mice. Our finding revealed that CR has different time dependent effect on the activity of mTOR complexes 1 and 2 and suggest that circadian clock protein BMAL1 is involved in the up regulation of mTORC2 upon CR in mammals.

## Introduction

Calorie restriction (CR) is a feeding regimen that increases longevity in organisms from yeast to primates [1]. The mechanisms of CR are not well known: multiple physiological and metabolic changes induced by CR have been reported and several signaling pathways are proposed mediators of CR effects. CR inhibits the activity of the mechanistic target of rapamycin (mTOR) [2,3]. mTOR is a kinase, which operates as two distinct complexes with different downstream targets and physiological functions, mTORC1 and mTORC2 [4,5]. Genetic or pharmacological inhibition of mTORC1 signaling leads to increased lifespan. It was proposed that high activity of mTORC1 is a major driving force of aging, while the suppression of mTOR contributes to many benefits of CR, including lifespan extension [6]. The suppression of mTORC1 activity by CR was demonstrated in several model organisms such as yeast, nematodes and flies, at the same time, studies conducted in mammals have not yet arrived at a consensus. Some observed suppression of mTORC1 signaling upon CR [7], while others detected no suppression and even induction in mTORC1 activity [8]. mTORC2 is also implicated in the control of aging, however, much less is known about its exact role in aging and the effect of CR on mTORC2 activity.

The activity of mTORC1 and mTORC2 is regulated by nutrients, hormones and growth factors [4]. In mammals, mTORC1 activity increases after feeding and reduces during fasting. We and others have recently reported that the activity of mTORC1 significantly oscillates during the day, and that this activity might be under the control of internal circadian clocks [9–14], in turn mTORC1 regulates the circadian clock by phosphorylating clock transcriptional factor BMAL1 [15,16]. BMAL1 was also implicated in the control of mTORC2 activity [17]. Circadian clocks are a network of interconnected molecular oscillators that generate 24-hour rhythms in gene expression and cell signaling, which is ultimately translated into the rhythms in behavior and physiology [18]. These rhythms contribute to the synchronization of metabolic processes in organisms with a periodic environment. Interestingly, CR and biological clocks might be interconnected: CR affects circadian rhythms in behavior, chromatin modifications and gene expression, and some of circadian clock proteins are involved in the CR mechanism [13,19–23]. During the caloric restriction procedure in mammals, food is provided once per day, at the same time every day. Animals are subject to a relatively short feeding period (3-4 hours), during which all outside nutrients are received, followed by a fasting period during which stored resources are consumed. mTOR signaling is known to respond to feeding and fasting [4,5], therefore the time of feeding and the time of tissue collection affect the mTOR complexes activity, and it might be critical for the correct interpretation of CR effects on mTOR signaling. The present study assayed activity of mTORC1 and mTORC2 across a daily cycle in wild type and circadian clock mutant mice. We found, in agreement with our expectation, that the CR effects on mTOR signaling are time-of-day dependent and some clock proteins might be involved in the response of mTOR complexes to CR.

## RESULTS

### CR regulates daily rhythms in mTORC1 activity in the liver

To assess the effect of CR on mTOR signaling, we compared daily rhythms mTORC1 and mTORC2 activities in the liver of mice that were subject to 30% CR for 2 months (CR group) with the activities in the liver of mice that had *Ad Libitum* (AL group) access to the food. CR mice received the food at ZT14 (two hours after light was off, ZT0 is the time when light is on and ZT12 is the time when light is off). We and others previously reported that feeding CR mice at ZT14 (or ZT12) did not significantly shift the phases of expression of core circadian clock genes [13,19–23] in the liver.

**Figure 1:**
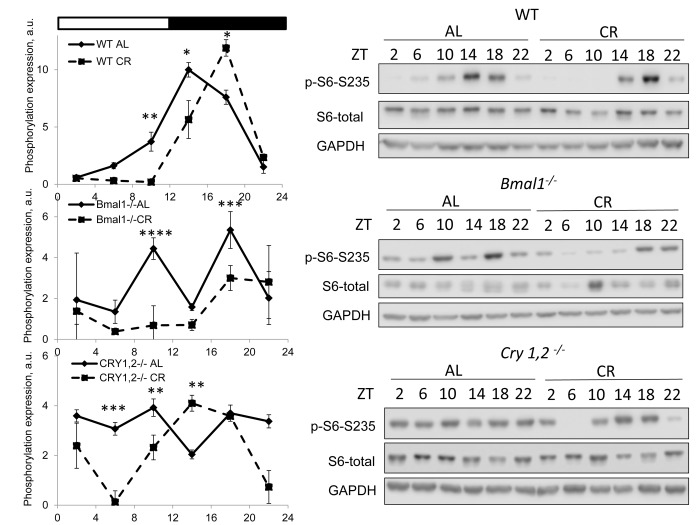
**CR affects TORC1 activity across the day independently from BMAL1 and CRYs.** Activity of mTORC1 in the liver of mice on AL and CR diets was assayed through phosphorylation of ribosomal protein S6 on Ser235/236. Representative WBs and quantification of diurnal rhythms in phosphorylation of S6 in wild type mice (upper panels), *Bmal1^-/-^* mice (middle panels) and *Cry1,2^-/-^* mice (lower panels). (AL) – black diamonds, black solid line, 30% caloric restriction (CR) – black squares and solid black lines. Two-way repeated ANOVA were used for statistical analysis, * - statistically significant difference (p<0.05) between diets (three male mice were used for every time point in both groups). Light and dark bars on the top of the figure represent light and dark phase of the day. ZT0 is the time when light is on and ZT12 is the time when light is off. Food for CR group was provided at ZT14. On the day of tissue collection at ZT14 tissues were harvested before mice received the food.

mTORC1 activity was assayed based on the phosphorylation of ribosomal protein S6 on S235/236 sites (S6 is not a direct target but it is often used as a surrogate marker of mTORC1 activity) [4,5] in the liver of wild type mice. First, we found significant variation in mTORC1 activity across the day for both AL and CR groups. As expected the activity was low during light phase the rest/fasting period and high during activity/feeding period (Figure 1A). The statistically significant effect of the diet on S6 phosphorylation was observed at ZT10, ZT14 (downregulation by CR) and ZT18 (upregulation by CR), at three other times the difference did not reach significance. Thus, we found that the effect of CR on mTORC1 activity depends on the time of the day. We also assayed the S6 phosphorylation in the liver of circadian mutant *Bmal1^-/-^* and *Cry1,2^-/-^* mice [24–26] under the same AL and CR conditions. For both mutant CR groups the levels of S6 phosphorylation were reduced during rest/fasting period similar to the effect of CR on wild type mice and it was induced at ZT14 in *Cry1,2^-/-^* mice but not in in *Bmal1^-/-^* mice. Thus, the circadian clock proteins BMAL1 and CRYs are not required for CR mediated down regulation of mTORC1 activity during rest/fasting period.

### CR regulates daily rhythms in mTORC2 activity in the liver

**Figure 2:**
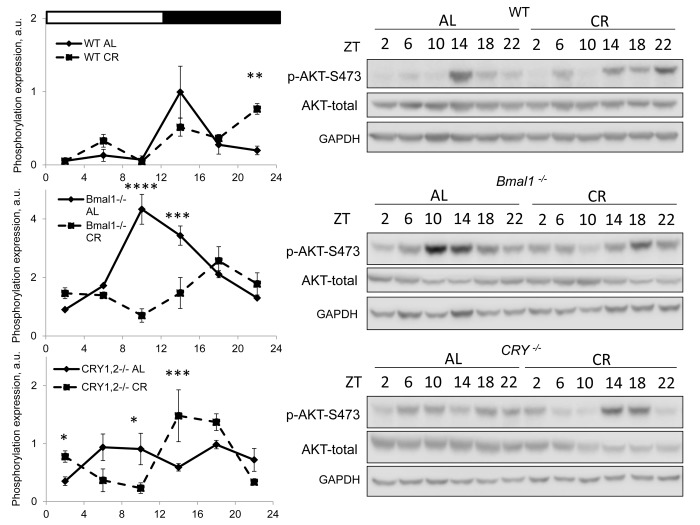
**CR effect on TORC2 activity is time of the day and BMAL1 dependent.** Activity of mTORC2 in the liver of mice on AL and CR diets was assayed through phosphorylation of AKT on S473. Representative WBs and quantification of diurnal rhythms in phosphorylation of AKT in wild type mice (upper panels), *Bmal1^-/-^* mice (middle panels) and *Cry1,2^-/-^* mice (lower panels). (AL) – black diamonds, black solid line, 30% caloric restriction (CR) – black squares and dashed black lines. Two-way repeated ANOVA were used for statistical analysis, * - statistically significant difference (p<0.05) between diets (three male mice were used for every time point in both groups). Light and dark bars on the top of the figure represent light and dark phase of the day. ZT0 is the time when light is on and ZT12 is the time when light is off. Food for CR group was provided at ZT14. On the day of tissue collection at ZT14 tissues were harvested before mice received the food.

We assayed mTORC2 activity through phosphorylation of its downstream target AKT on S473 [27]. Results are presented on Figure 2. Similar to the mTORC1, the activity of mTORC2 was different at different times on both diets. Phosphorylation of AKT-S473 was high in the liver of wild type mice at ZT14-18 for AL group and it was high at ZT14-22 and ZT6 for CR group. The difference between diets was significant at ZT22, the phosphorylation of AKT was higher in the liver of CR mice. Thus, CR affected the activities of mTORC1 and mTORC2 differently, by decreasing the mTORC1 activity at several times and increasing the mTORC2 activity in wild type mice. AKT phosphorylation also demonstrated strong daily fluctuation in the liver of AL *Bmal1^-/-^* mice with high level at ZT10-18. In contrast to the effect in wild type mice CR did not induce mTORC2 activity but resulted in significant reduction at ZT10-14. mTORC2 responded differently to CR in the liver *Cry1,2^-/-^* mice: the phosphorylation of AKT-S473 was reduced at ZT6/ZT10 and increased at ZT14/ZT18. Thus, circadian clock protein BMAL1 but not CRYs is important for the CR mediated induction in mTORC2 activity.

**Figure 3:**
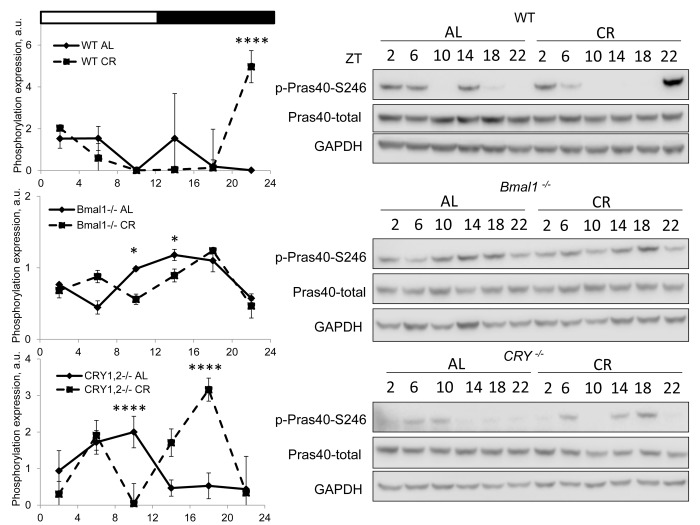
**CR effect on AKT activity is time of the day and BMAL1 dependent.** Activity of AKT in the liver of mice on AL and CR diets was assayed through phosphorylation of PRAS on S246. Representative WBs and quantification of diurnal rhythms in phosphorylation of PRAS in wild type mice (upper panels), *Bmal1^-/-^* mice (middle panels) and *Cry1,2^-/-^* mice (lower panels). (AL) – black diamonds, black solid line, 30% caloric restriction (CR) – black squares and dashed black lines. Two-way repeated ANOVA was used for statistical analysis, * - statistically significant difference (p<0.05) between diets (three male mice were used for every time point in both groups). Light and dark bars on the top of the figure represent light and dark phases of the day. ZT0 is the time when light is on and ZT12 is the time when light is off. Food for CR group was provided at ZT14. On the day of tissue collection at ZT14 tissues were harvested before mice receive the food.

TORC2-dependent phosphorylation of AKT stimulates the kinase activity, and we decided to check the effect of CR on the phosphorylation of PRAS40 at S246, it is known that this site is phosphorylated by AKT [28]. We assayed the phosphorylation in wild type and mutant mice under both diets (Figure 3). PRAS40 phosphorylation was increased in wild type at ZT22 and was reduced in the liver of *Bmal1^-/-^* at ZT10 and ZT14. The phosphorylation was increased at ZT18 and decreased at ZT10 in *Cry1,2^-/-^* mice. Thus, we observed a good correlation between AKT and PRAS40 phosphorylation.

## DISCUSSION

We reported here that CR affected daily changes in mTORC1 and mTORC2 activity in the liver. mTOR plays an important role in the control of metabolism and aging. High mTORC1 activity promotes aging and suppression of mTORC1 increases longevity. mTORC2 might also be involved in the control of longevity but, in contrast to mTORC1, it might be a positive regulator of longevity [29]. Changes in mTORC1 activity are implicated in the mechanism of CR [30]. It was previously reported that CR affects activity of mTORC1: data in invertebrate model organisms are consistent [31] but published results in the rodents were not in agreement with each other [7,8]. Our data might provide an explanation to this discrepancy, indeed, depending on the time of the analysis, CR might result in decreased, increased or no changes in activity of mTORC1. Little is known about the influence of CR on mTORC2 activity. We found the effect of CR on mTORC2 signaling was also time-of-day dependent.

It is important to mention that feeding is a regulator of mTOR activity and CR can change a feeding pattern, therefore, some of the observed effects can be due to the changes in the feeding. It is also well documented that phases of clock gene expression in the liver are strongly affected by time of the feeding [32]. Feeding profile of wild type mice on AL and CR diets was recently carefully recorded [23], AL mice consumed the food predominantly during the dark phase with maximum consumption between ZT14 and ZT18. In our study we provided the food for CR group at ZT14, and mice consumed most of the food between ZT14 and ZT18. Therefore, we expect that selected feeding schedule will disturb the circadian clock in the liver in most minimal way. Indeed, as it was reported, feeding CR mice at ZT14 [19,20] or at ZT12 [13] has minimal or no effect on phases of clock gene expression in the liver. Thus, we concluded that feeding at ZT14 is the most appropriate time to study the effect of CR in wild type mice. The behavior of circadian clock mutant mice is rhythmic under 12:12 light/dark cycle, but feeding behavior might be affected by clock disruption, therefore, different pattern of mTOR activity between wild type and mutants under AL might be due to the difference in the feeding profile, but under CR diets all wild type and mutants have the same feeding profile, they consumed the food between ZT14 and ZT18. However, the daily profiles of mTORC1 and mTORC2 activities under CR were not identical between wild type and mutants: for example, the peak of mTORC1 under CR was observed at ZT18 in wild type, at ZT18 in *Bmal1^-/-^* and at ZT14 in *Cry1,2^-/-^* mice; mTORC2 activity was high at ZT22 in wild type, intermediate in *Bmal1^-/-^* and low in *Cry1,2^-/-^* mice. Together that suggests that the change in feeding pattern is not the only regulator of CR induced effects on mTOR signaling.

Difference in the amount of the food consumption between wild type and mutants might be another contributing factor. There is no significant difference in food consumption between wild type *Bmal1^-/-^* mice [20], while *Cry1,2^-/-^* mice consume about 15% less food [33]. We compared total daily activity of mTORC1 and mTORC2 under CR and AL. We found that average activity of mTORC1 was reduced in all three genotypes and average activity of mTORC2 was increased in wild type, reduced in *Bmal1^-/-^* and did not changed in *Cry1,2^-/-^* mice. Thus, the difference in the response of mTOR complexes to CR between wild type and mutants cannot be explained through the difference in total food intake: wild type and *Bmal1^-/-^* mice have the same food intake but different responses.

mTORC1 and mTORC2 share the same catalytic subunit, the TOR kinase [4,5]. It was postulated that the TOR kinase is a limiting factor and complexes compete for it [34]. In addition, the complexes regulate each other activity through phosphorylation of the complexes components [35,36]. We used JTK analysis to assay if the activities of mTOR complexes have circadian rhythms. The result of rhythms analysis is presented in Supplementary Table 1. mTORC1 activity was rhythmic for wild type at both diets, both mutants were arrhythmic under AL and *Bmal1^-/-^* became rhythmic under CR, while *Cry1,2^-/-^* did not. mTORC2 activity was rhythmic under AL in wild type and *Bmal1^-/-^* mice, it lost the rhythms in wild type under CR but it was still rhythmic in *Bmal1^-/-^* mice. The peaks in mTORC1 and mTORC2 activities coincided at ZT14 for AL wild type mice, while mTORC1 peaked at ZT 16 and mTORC2 peaked at ZT 20 (Supplementary Table 1) in CR wild type mice. Thus, under CR the peaks in mTORC1 and mTORC2 activities were temporally separated in wild type mice. No such temporal separation between peaks was observed in circadian clock mutants: the peaks are at ZT 18 (mTORC1) and ZT 20 (mTORC2) in the liver of *Bmal1^-/-^* mice and at ZT18 (for both) in the liver of *Cry1,2^-/-^*mice. Interestingly, at wildness, animals do not have continuous access to the food and our data suggest that intact circadian clock is necessary for temporal separation of the peaks in mTORC1 and mTORC2 activities under conditions of limited nutrients supply.

Based on the presented data, we proposed the following hypothesis: the balance between the complexes established under AL feeding was changed by CR and shifted toward mTORC2. Circadian clock and circadian clock proteins contributed to this shift through compartmentalization of mTORC1 and mTORC2 activities in time. The effect of CR was further compromised in *Bmal1^-/-^* mice, suggesting that BMAL1 has some clock independent functions in CR. Indeed, CR regulated expression and activity of the clock transcriptional factor BMAL1 is necessary for the regulation of CR-controlled signaling pathways and for the full benefits of CR. Interestingly, while *Bmal1^-/-^* mice demonstrate multiple metabolic abnormalities as well as accelerated aging and reduced lifespan [37], this phenotype can be partially reversed by treatment with pharmacological inhibitor of mTOR activity – rapamycin [10]. BMAL1 is a negative regulator of mTORC1 [10,14] and a positive regulator of mTORC2 [17] activities under AL, according to our findings BMAL1 was important for the up regulation of mTORC2 and was not important for the down regulation of mTORC1 under CR. In summary, we report differential regulation of mTORC1 and mTORC2 by CR and involvement of the circadian clock in this regulation. These results encourage further studies on the role of biological clocks and clock proteins in CR and open an opportunity to use clock mechanisms to improve or even mimic CR.

## METHODS

### Animals

Wild type and circadian mutant mice were on C57BL/6J background. *Bmal1^-/-^* and *Cry1,2^-/-^* mice were previously described [24,25]. Animals were maintained on a 12:12 light:dark cycle with lights on at 7:00 am, and fed 18% protein rodent diet (Harlan). *Ad libitum* (AL) group had unrestricted access to food. Animals on Caloric restriction (CR) diet received their food once per day at ZT14. CR was started at 3 months of age. Animals were on 30% CR for two months before tissue collection. All groups had unrestricted access to water. All tissue collection experiments were performed for 5-month-old wild type, *Bmal1^-/-^* and *Cry1,2^-/-^* mice. All animal studies were conducted in accordance with the regulations of the Committee on Animal Care and Use at Cleveland State University.

### Analysis of protein phosphorylation and expression

Analysis of protein expression was performed on liver tissues collected every four hours around the clock and stored at -80^o^C. For quantitative analysis, liver samples were run individually to estimate variability between biological replicates. For a representative WB, liver lysates from three different mice were pooled together for each time point. Lysates were prepared with lysis buffer with Protease/Phosphatase Inhibitor Cocktail (Cell Signaling Technology, Beverly, MA, USA) using a sonicator. Protein concentration was determined using Bradford protein assay kit. Protein loading was checked by Ponceau staining. List of primary antibodies is in Supplementary Table 2. Probing the same membranes with anti-GAPDH antibodies was used for normalization of the signal. Images were obtained with LI-COR, Lincoln, NE, quantification was performed with the Image Studio Lite version 5.2 software.

### Statistical analysis

At least 3 animals for every time point, for each feeding type and for each genotype were used for all experiments. Data are shown as average +/- standard deviation. IBM SPSS Statistics 20 and GraphPad software packages were used for analysis. The effect of diet (AL versus CR) and time of day were tested for significance with two-way repeated ANOVA corrected for multiple comparison using Bonferroni. P<0.05 was considered as statistically significant difference.

## Supplementary Material

Supplementary Table 1

Supplementary Table 2
