# Supplementary material for: Caloric restriction effects on liver mTOR signaling are time-of-day dependent
Source: Aging (Albany NY). 2018 Jul 16;10(7):1640–8. doi: 10.18632/aging.101498 (PMC6075448; doi:10.18632/aging.101498)
Supplement: Supplementary Table 1 [file aging-10-101498-s001.pdf]

Supplementary Table 1. Analysis of circadian rhythms by JTK software.

	P value	Phase	Circadian
S6-S235 AL WT	3.7e-07	14	YES
S6-S235 CR WT	6.4e-06	16	YES
S6-S235 *Bmal1*$^{-/-}$ AL	0.45	16	NO
S6-S235 *Bmal1*$^{-/-}$ CR	1.1e-05	18	YES
S6-S235 *Cry1,2*$^{-/-}$ AL	0.14	2	NO
S6-S235 *Cry1,2*$^{-/-}$ CR	0.74	18	NO
AKT-S473 AL WT	0.04	14	Yes
AKT-S473 CR WT	0.45	20	No
AKT-S473 *Bmal1*$^{-/-}$ AL	0.001	10	Yes
AKT-S473 *Bmal1*$^{-/-}$ CR	0.02	20	Yes
AKT-S473 *Cry1,2*$^{-/-}$ AL	1	10	No
AKT-S473 *Cry1,2*$^{-/-}$ CR	0.31	18	No
Pras40-S246 AL WT	1	6	No
Pras40-S246 CR WT	0.01	22	Yes
Pras40-S246 *Bmal1*$^{-/-}$ AL	0.0002	14	Yes
Pras40-S246 *Bmal1*$^{-/-}$ CR	0.26	14	No
Pras40-S246 *Cry1,2*$^{-/-}$ AL	0.01	10	Yes
Pras40-S246 *Cry1,2*$^{-/-}$ CR	1	18	No
