# Supplementary material for: Caloric restriction effects on liver mTOR signaling are time-of-day dependent
Source: Aging (Albany NY). 2018 Jul 16;10(7):1640–8. doi: 10.18632/aging.101498 (PMC6075448; doi:10.18632/aging.101498)
Supplement: Supplementary Table 2 [file aging-10-101498-s002.pdf]

Supplementary Table 2. List of antibodies used for the Western blot analysis.

Antibody	Catalog No.	Company
P-S6 (S235/236)	2211	CST
Total S6	J1416	Santa Cruz
P-Akt (S473)	4060	CST
Total- AKT	4691	CST
P-Pras40 (T246)	13175	CST
Total-Pras40	2691	CST
GAPDH	5174	CST
